# ID 132 - Custo-Utilidade do Mepolizumabe em Crianças e Adolescentes com Asma Eosinofílica Grave Refratária no Sistema Único de Saúde (SUS)

**DOI:** 10.5327/2237-9622.2025.v34s1.132

**Published:** 2025-11-25

**Authors:** Ivan Ricardo Zimmermann, Flávia Tavares Elias, Erica Tatiane da Silva

**Keywords:** mepolizumabe, asma, custo-efetividade, Sistema Único de Saúde

## Abstract

**Introdução:**

A asma é a doença crônica mais comum entre crianças e impacta todas as faixas etárias. No Brasil, o SUS reembolsa o uso de mepolizumabe como tratamento adicional para adultos com asma eosinofílica grave. Este estudo visa avaliar o custo-efetividade da ampliação do reembolso para pacientes a partir de 6 anos.

**Método:**

O estudo definiu como população os pacientes a partir de 6 anos de idade que são refratários ao tratamento com corticosteroide inalatório (CI) combinado com beta-agonistas de longa duração (LABA). Usando um modelo de Markov com ciclos mensais, analisamos as transições entre os estados de saúde sem exacerbação, com exacerbação (incluindo uso de corticoesteroides orais, admissão na emergência e hospitalização) e morte. Custos médicos diretos foram estimados com base nas fontes oficiais do SUS (BPS e Sigtap) e o principal desfecho em saúde foi o QALY. Foram realizadas análises de sensibilidade determinísticas e probabilísticas com o Microsoft Excel®.

**Resultados:**

No caso base, considerando o preço atual do mepolizumabe reembolsado pelo Ministério da Saúde (R$ 4.756,28 por frasco de 100 mg), a razão incremental de custo-efetividade (RCEI) foi de R$ 671.899,62/QALY, excedendo o limiar de R$ 40.0000,00 a R$ 120.000,00 proposto pela Comissão Nacional de Incorporação de Tecnologias (Conitec). Com o preço médio de compras públicas locais (R$ 2.060,05), a RCEI foi de R$ 476.735,74/QALY. Considerando um desconto anterior proposto pelo fabricante (R$ 1.927,81) e fracionamento de doses para crianças até 12 anos, o ICER foi estimado em R$ 249.400,93/QALY.

**Conclusão:**

Os resultados indicam que, a preços de reembolso atuais, o uso de mepolizumabe em crianças e adolescentes não é custo-efetivo. Cenários alternativos com descontos nos preços e fracionamento de doses podem alterar essas conclusões. Comparando com outros estudos, os achados destacam a necessidade de negociações de preço para aprimorar o acesso ao tratamento.

